# Polymerase Chain Reaction-Lateral Flow Strip for Detecting *Escherichia coli* and *Salmonella enterica* Harboring *bla*_CTX-M_

**DOI:** 10.3390/antibiotics14080745

**Published:** 2025-07-24

**Authors:** Rujirat Hatrongjit, Sumontha Chaisaeng, Kulsatree Sitthichotthumrong, Parichart Boueroy, Peechanika Chopjitt, Ratchadaporn Ungcharoen, Anusak Kerdsin

**Affiliations:** 1 Department of General Science, Faculty of Science and Engineering, Kasetsart University Chalermphrakiat, Sakon Nakhon Campus, Sakon Nakhon 47000, Thailand; 2 Special Research Unit of Emerging Foodborne Pathogens Surveillance, Kasetsart University, Bangkok 10900, Thailand; sumonthachaisaeng@gmail.com (S.C.); parichart.bou@ku.th (P.B.); peechanika.c@ku.th (P.C.); 3 Faculty of Public Health, Kasetsart University Chalermphrakiat, Sakon Nakhon Campus, Sakon Nakhon 47000, Thailand; kulsatri69earth@gmail.com (K.S.); ratchadaporn.un@ku.th (R.U.)

**Keywords:** cephalosporins, *bla*_CTX-M_, nucleic acid amplification, lateral flow strip, *Escherichia coli*, *Salmonella*

## Abstract

**Background:** *Salmonella enterica* and *Escherichia coli* are common foodborne pathogens of global concern, particularly due to their antimicrobial resistance, notably to cephalosporins. **Objective:** This study aimed to evaluate a polymerase chain reaction-based lateral flow strip (PCR-LFS) assay for the detection of *Salmonella* spp. and *E. coli* harboring the *bla*_CTX-M_ gene, which confers resistance to third-generation cephalosporins. **Methods:** Two duplex PCRs (dPCR) were established to detect *E. coli*-harboring *bla*_CTX-M_ (set 1) and *Salmonella*-harboring *bla*_CTX-M_ (set 2). 600 bacterial isolates and raw pork mince spiked with *bla*_CTX-M_-harboring *E. coli* and *Salmonella* were used to evaluated. **Results:** Both dPCR assays successfully detected *bla*_CTX-M_-positive *E. coli* or *Salmonella* strains, while strains lacking the gene showed no amplification. Non-*E. coli* and non-*Salmonella* strains were PCR-negative unless they carried *bla*_CTX-M_. The dPCR-LFS showed 100% validity including accuracy, sensitivity, specificity, positive predictive value, and negative predictive value for both *E. coli* or *Salmonella* spp. harboring or lacking *bla*_CTX-M_. The assay accurately detected target strains without cross-reactivity with other bacteria or antimicrobial resistance genes. Cohen’s Kappa coefficient indicated perfect agreement (*κ* = 1), reflecting the high reliability of the dPCR-LFS. The assay could detect as low as 25 CFU/mL for *bla*_CTX-M_-positive *E. coli* and 40 CFU/mL for *bla*_CTX-M_-positive *Salmonella* in spiked raw pork mince. **Conclusions:** This assay is rapid, easy to interpret, and suitable for large-scale screening in surveillance programs.

## 1. Introduction

Foodborne pathogens in contaminated food are a significant concern in food safety and require close monitoring. These pathogens include viruses, bacteria, fungi, and protozoa [1]. Generally, infection occurs through the consumption of food or water contaminated with foodborne pathogens or their toxins. Detection and control of foodborne pathogens are prerequisites for protecting human and animal health and maintaining international trade. Rapid detection of foodborne pathogens is key to effective surveillance systems, ensuring a safe food supply, and preventing foodborne diseases [2,3,4].

Among these foodborne pathogens, *Salmonella enterica* and *Escherichia coli* have been implicated in numerous foodborne diseases associated with the consumption of contaminated food and water [1]. These bacterial pathogens are critical targets for the monitoring and surveillance of both phenotypic and genotypic antimicrobial resistance (AMR) in food-producing animals, fresh and processed meat, vegetables, and drinking water. Additionally, *Salmonella*, *E. coli*, *Campylobacter* spp., and *Enterococcus* spp. are recommended as indicators for AMR surveillance in human and non-human sectors (i.e., food supply chains, animals, and the environment) [5,6,7].

The emergence of extended-spectrum β-lactamase (ESBL)-producing bacteria has conferred resistance to penicillin and third- and fourth-generation cephalosporins, which are critically important antibiotics in both human and veterinary medicine [8]. These pathogens are recognized by the World Health Organization (WHO) as among the most challenging to treat [9]. Infections caused by ESBL-producing bacteria in humans and animals have been widely reported, with documented transmission routes including food, water, and contact with contaminated environments [10]. Thus, monitoring ESBL-producing *Salmonella* spp. and *E. coli* is essential for public health.

ESBL has been classified into three main groups, including Ambler class A ESBL, miscellaneous ESBL, and ESBL that degrade carbapenems [11]. Most ESBLs belong to Ambler class A, which includes sulfhydryl reagent variable (SHV), Temoniera (TEM), and cefotaxime-M (CTX-M) types [11]. Among these, CTX-M-type ESBLs are the most widely distributed globally [12,13,14]. CTX-M type-ESBLs have increased in prevalence since 2000, and this is an important mechanism for developing resistance to cephalosporins, posing a major difficulty in clinical treatment, with restricted options to treat infections caused by CTX-M-producing bacteria [12,14]. This trend has led to increased reliance on carbapenems [12]. CTX-M-producing bacteria are now frequently detected in Europe, Asia, and the Americas [14]. Over 80 CTX-M variants have been reported across hospitals, communities, food animals, fresh produce, water, and the environment [15].

Routine screening and rapid detection of ESBL-producing bacteria in clinical laboratory settings are critical for infection control and therapeutic decision-making. The Clinical and Laboratory Standards Institute (CLSI) recommends a two-step approach for ESBL detection [16]. Initial screening methods include the Kirby–Bauer disk diffusion test and automated systems such as Vitek. Confirmatory tests involve the double-disk synergy test (DDST), combination disk methods, or E-test ESBL strips. In addition to phenotypic confirmatory methods, genotypic assays such as polymerase chain reaction (PCR) and nucleotide sequencing are employed to detect specific ESBL-encoding genes and their variants [11]. Other molecular methods include isoelectric point determination, DNA probes, oligotyping, PCR-restriction fragment length polymorphism (PCR-RFLP), PCR-single-strand conformational polymorphism (PCR-SSCP), real-time PCR, matrix-assisted laser desorption ionization–time of flight mass spectrometry (MALDI-TOF), and the NG-Test CTX-M MULTI, a rapid immunochromatographic lateral flow strip assay (LFS) [11].

Among these techniques, nucleic acid amplification followed by LFS, such as PCR-LFS and recombinase polymerase amplification (RPA)-LFS, is simple and widely applied for detecting various pathogens and antimicrobial resistance genes [17,18,19,20,21,22]. LFS is a paper-based device that uses colloidal gold nanoparticles as signal labels [23]. Combining PCR or RPA with LFS improves laboratory efficiency and enables rapid visualization of target genes, eliminating the need for gel electrophoresis and staining, thereby reducing detection time and processing complexity. The principles of PCR-LFS or RPA-LFS involve labeling the 5′ end of the forward primer with fluorescein isothiocyanate (FITC) and the 5′ end of the reverse primer with biotin or digoxigenin, followed by amplification using PCR or RPA [21,24]. The labeled amplicons bind to specific antibodies on the test line of the colloidal gold strip, producing a visual colorimetric signal interpretable by the naked eye.

*Salmonella* spp. and *E. coli* are important indicator organisms for pathogen and AMR surveillance, particularly in relation to cephalosporin nonsusceptibility due to *bla*_CTX-M_. Although many PCR assays have been developed to detect *Salmonella* spp. and *E. coli* harboring antimicrobial resistance genes such as *bla*_CTX-M_ [25,26], neither PCR-LFS nor RPA-LFS currently allow simultaneous detection of both bacterial species and resistance genes in a single assay [18,27,28,29]. In this study, we evaluated a polymerase chain reaction-based lateral flow strip (PCR-LFS) assay for detecting *Salmonella* spp. and *E. coli* harboring *bla*_CTX-M_, aimed at infection control and surveillance in environmental, livestock, food, or feed contamination, particularly in resource-limited laboratories. This assay is rapid, easy to perform and interpret, and suitable for high-throughput screening in AMR surveillance programs.

## 2. Results

**Figure 1:**
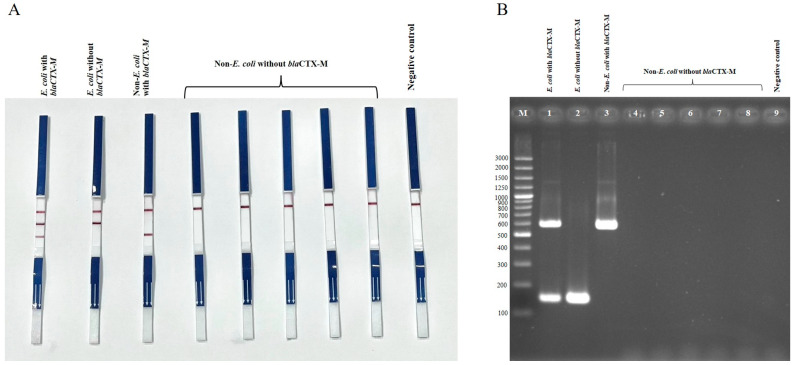
Duplex PCR-LFS (**A**) and duplex PCR–gel electrophoresis (**B**) detection of *E. coli* with and without *bla*_CTX-M_, and non-*E. coli* with and without *bla*_CTX-M_. (**A**) Top line = control line; middle line = *E. coli*; bottom line = *bla*_CTX-M_. *E. coli* carrying *bla*_CTX-M_ shows two bands (species-specific and *bla*_CTX-M_) (first strip from left). *E. coli* lacking *bla*_CTX-M_ shows only the *E. coli* line (second strip). Non-*E. coli* strains carrying *bla*_CTX-M_ show only the *bla*_CTX-M_ line (third strip). Non-*E. coli* strains without *bla*_CTX-M_ show no target lines (strips 4–8). Negative control = *Klebsiella pneumoniae* ATCC 13883. (**B**) Lane M = 100 bp DNA ladder; Lane 1 = *E. coli* with *bla*_CTX-M_; Lane 2 = *E. coli* without *bla*_CTX-M_; Lane 3 = non-*E. coli* with *bla*_CTX-M_; Lanes 4–8 = non-*E. coli* without *bla*_CTX-M_; Lane 9 = negative control (*Klebsiella pneumoniae* ATCC 13883).

**Figure 2:**
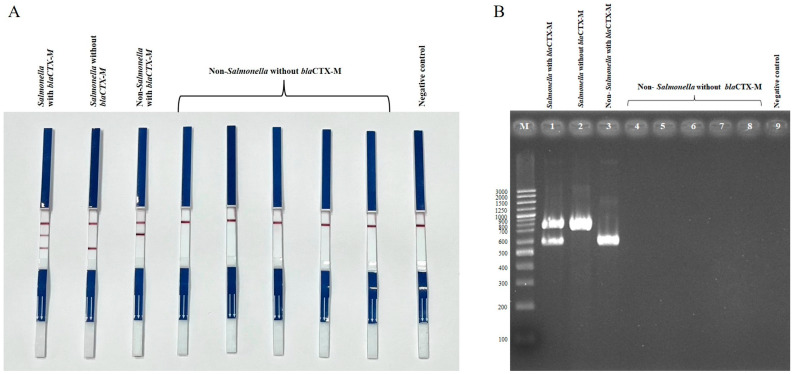
Duplex PCR-LFS (**A**) and duplex PCR-gel electrophoresis; (**B**) detection of *Salmonella* spp. with and without *bla*_CTX-M_, and non-S*almonella* spp. with and without *bla*_CTX-M_. (**A**) Top line = control line; middle line = *bla*_CTX-M_; bottom line = *Salmonella*. *Salmonella* carrying *bla*_CTX-M_ shows two lines (first strip). *Salmonella* without *bla*_CTX-M_ shows only the species-specific line (second strip). Non-*Salmonella* carrying *bla*_CTX-M_ shows the *bla*_CTX-M_ line (third strip). Non-*Salmonella* without *bla*_CTX-M_ shows no target lines (strips 4–8). Negative control = *Klebsiella pneumoniae* ATCC 13883. (**B**) Lane M = 100 bp DNA ladder; Lane 1 = *Salmonella* with *bla*_CTX-M_; Lane 2 = *Salmonella* without *bla*_CTX-M_; Lane 3 = non-*Salmonella* with *bla*_CTX-M_; Lanes 4–8 = non-*Salmonella* without *bla*_CTX-M_; Lane 9 = negative control (*Klebsiella pneumoniae* ATCC 13883).

Two duplex PCR (dPCR) assays were established to detect *E. coli* harboring *bla*_CTX-M_ (set 1) and *Salmonella* harboring *bla*_CTX-M_ (set 2). Both assays successfully detected *bla*_CTX-M_-positive *E. coli* and *Salmonella* strains, while *E. coli* and *Salmonella* strains lacking *bla*_CTX-M_ showed no *bla*_CTX-M_ amplification, and the negative control (*Klebsiella pneumoniae* ATCC13883) did not show any bands (Figure 1 and Figure 2). Non-*E. coli* and non-*Salmonella* strains tested negative in the dPCR assays unless they carried *bla*_CTX-M_, in which case only the *bla*_CTX-M_ band was detected. For bacterial species other than *E. coli*, *Salmonella*, or Enterobacterales that do not harbor *bla*_CTX-M_, the PCR-LFS produced negative results for both target bands, while the internal strip-control band remained positive. These results confirm the high specificity of the duplex PCR-LFS for *E. coli* and *Salmonella*.

The PCR-LFS assay was evaluated using 200 *E. coli* isolates and 30 *Salmonella* isolates harboring *bla*_CTX-M_ (Table 1). All isolates showed positive results for both the species-specific and *bla*_CTX-M_ bands. *E. coli* and *Salmonella* strains carrying other antimicrobial resistance genes (but not *bla*_CTX-M_) showed amplification of only the species-specific band. In contrast, *bla*_CTX-M_*-*harboring strains of other species (e.g., *Klebsiella pneumoniae*, *Enterobacter cloacae*, *Citrobacter freundii*) showed positive results only for the *bla*_CTX-M_ band. Sanger sequencing of representative amplicons confirmed the identities of *E. coli*, *Salmonella*, and *bla*_CTX-M_. Non-*Enterobacterales* species tested negative for all targets, indicating no cross-reactivity.

**Figure 3:**
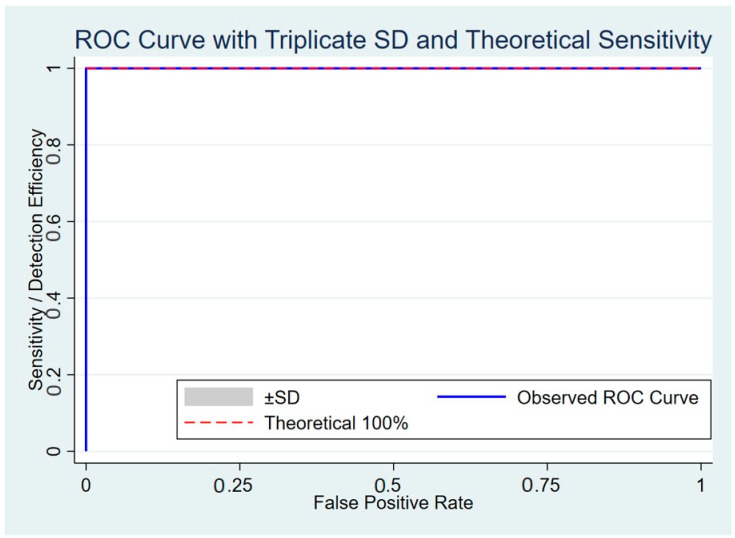
ROC curve showing triplicate standard deviation and theoretical sensitivity of duplex PCR-LFS detecting *E. coli* and *Salmonella* carrying *bla*_CTX-M_.

As shown in Table 2, Table 3 and Table 4, the dPCR-LFS assay demonstrated 100% validity, including accuracy, sensitivity, specificity, positive predictive value, and negative predictive value, for both *E. coli* and *Salmonella* strains harboring or lacking *bla*_CTX-M_. The assay accurately detected the target species without cross-reactivity with non-target bacteria or other antimicrobial resistance genes. Cohen’s kappa coefficient indicated perfect agreement (*κ* = 1), confirming the high reliability of the dPCR-LFS. Statistical analysis revealed a significant association (*p* < 0.01) for true positives. The coefficient of determination (R^2^) was 0.92952 for simultaneous detection of *E. coli* and *Salmonella* with *bla*_CTX-M_, and 0.96621 and 0.96642 for detecting either target species or *bla*_CTX-M_ alone, respectively. A receiver operating characteristic (ROC) curve confirmed that the assay’s sensitivity and specificity were consistent with theoretical expectations (Figure 3).

**Figure 4:**
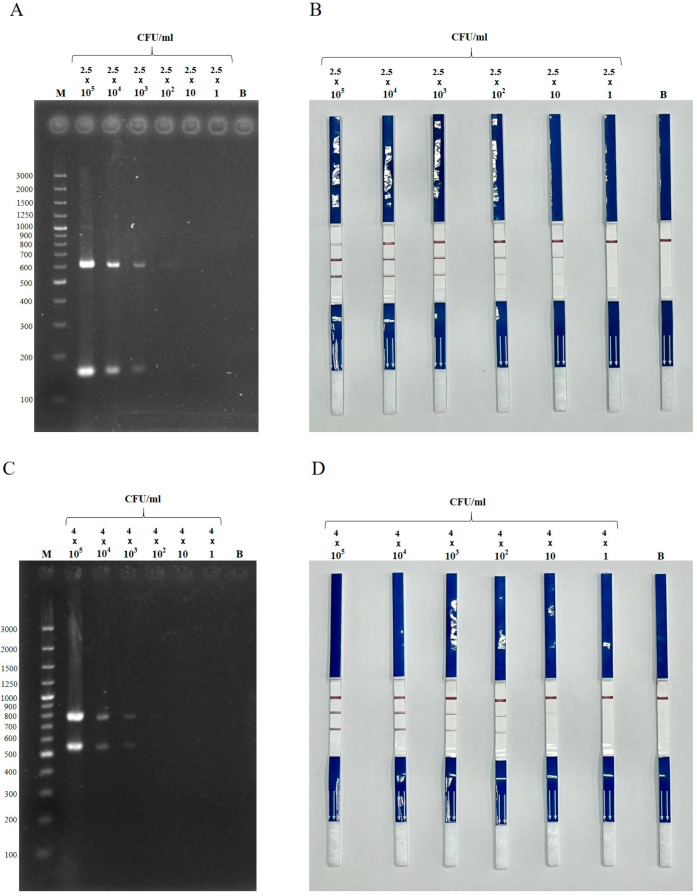
Detection limit of duplex PCR–gel electrophoresis (**A**,**C**) and duplex PCR-LFS (**B**,**D**) detecting *E. coli* (**A**,**B**) and *Salmonella* spp. (**C**,**D**) harboring *bla*_CTX-M_, spiked in raw pork mince. Dilutions: 2.5 × 10^5^ to 2.5 CFU/mL for *E. coli*, and 4 × 10^5^ to 4 CFU/mL for *Salmonella*. M = 100 bp DNA ladder; B = blank control (uninoculated).

The limit of detection of the duplex PCR-LFS assay was 72 fg and 105 fg of genomic DNA for *E. coli* and *Salmonella*, respectively. In the case of *bla*_CTX-M_ detection, the assay identified as little as 7.2 pg for *E. coli* carrying *bla*_CTX-M_ and 105 fg for *Salmonella* carrying *bla*_CTX-M_. When tested in artificially spiked raw pork mince, the assay could detect as low as 25 CFU/mL for *bla*_CTX-M_-positive *E. coli* and 40 CFU/mL for *bla*_CTX-M_-positive *Salmonella* (Figure 4). However, the signal at these lowest concentrations was noticeably weaker compared to 10^2^ CFU/mL. In contrast, dPCR–gel electrophoresis had a detection limit of 10^3^ CFU/mL for both targets. The duplex PCR-LFS assay was more time-efficient than PCR followed by gel electrophoresis. Excluding DNA extraction, the cost per sample for dPCR-LFS was approximately USD 2.25–2.45 per set. The total running time for dPCR-LFS was approximately 60–70 min.

## 3. Discussion

In 2017, the WHO released guidance on the integrated surveillance of antimicrobial resistance (AMR) in foodborne bacteria, recommending a One Health approach and suggesting the monitoring of *Salmonella*, *Campylobacter* spp., *E. coli*, and *Enterococcus* spp. as priority organisms [6]. Similarly, in 2019, the Food and Agriculture Organization (FAO) designated the same bacterial species as targets for AMR surveillance [5]. In 2021, the WHO introduced the Tricycle Surveillance Program, which emphasizes the monitoring of ESBL-producing *E. coli* across human, food supply chain, and environmental sectors [7]. These organisms serve as key indicators in global AMR surveillance systems.

Rapid diagnostic methods are critical to reducing the spread of antimicrobial-resistant bacteria, enabling timely isolation and appropriate treatment. A variety of rapid detection technologies have been developed, including nucleic acid amplification (NAA) techniques, immunochromatographic tests, electrochemical assays, microarrays, nanoparticle-based systems, and mass spectrometry [30,31,32,33]. Among these, NAA-based methods such as PCR, real-time PCR, loop-mediated isothermal amplification (LAMP), and recombinase polymerase amplification (RPA) are particularly favored. These have been applied for the detection of *Salmonella* spp., *E. coli*, *Klebsiella pneumoniae*, and other Enterobacterales harboring *bla*_CTX-M_ or other antimicrobial resistance genes [18,34,35,36,37].

In this study, two sets of duplex PCR-based lateral flow strip (dPCR-LFS) assays were evaluated to detect *E. coli*, *Salmonella*, and *bla*_CTX-M_ for use in surveillance of foods, the environment, livestock, and hospital infection control purposes. The method is rapid and user-friendly, with results easily interpreted without the need for gel electrophoresis or specialized visualization instruments. These characteristics make it suitable for application in both human and non-human sectors, especially in the food safety program part of surveillance.

NAA-based lateral flow assays (NAA-LFS) have been developed for detecting various pathogens, including *Vibrio parahaemolyticus*, *E. coli*, *Salmonella*, *Bacillus anthracis*, *Yersinia pestis*, *Listeria monocytogenes*, *Staphylococcus aureus*, and coliforms [20,38,39,40,41,42,43,44]. For example, mPCR-LFS assays have enabled the simultaneous detection of *E. coli* O157:H7 and *S. Typhimurium* [45]. PCR-LFS has also been applied for detecting *E. coli*, coliform bacteria, and total bacterial load [42]. A lateral flow biosensor based on LAMP-CRISPR/Cas12a has been developed for *Salmonella* detection in food samples [46]. However, few NAA-LFS assays enable simultaneous detection of both bacterial species and resistance genes, as demonstrated in this study. For example, RPA-LFS assays have detected antimicrobial resistance genes such as *bla*_CTX-M_, *bla*_SHV_, *bla_OXA_*, *bla_KPC_*, *bla_NDM_*, *mcr-1*, and *tet(X4)*, but without identifying the bacterial species carrying them [18,37,47,48]. One prior study used mPCR-LFS to detect *bla_KPC_* and *bla_NDM_* in Enterobacterales [49].

A comparison of detection limits between dPCR-LFS and dPCR–gel electrophoresis, using raw pork mince spiked with *E. coli* and *S. enterica* harboring *bla*_CTX-M_, showed that dPCR-LFS had a lower detection limit. Our dPCR-LFS assay detected bacterial concentrations as low as 25–40 CFU/mL, consistent with previous findings demonstrating low detection limits for NAA-LFS in food matrices [45,50,51,52]. For instance, a previous study reported that mPCR-LFS detected *E. coli* O157:H7 and *S*. Typhimurium in spiked cabbage at limits of 10^4^ CFU/25 g and 10^3^ CFU/25 g, respectively [45]. Zeng et al. (2020) reported a detection limit of 50 CFU/mL for *Vibrio parahaemolyticus* using PCR-LFS [52]. Another study detected methicillin-resistant *Staphylococcus aureus* at 200 CFU/100 g of pork products using PCR-LFS [53]. Wang et al. (2020) showed that RPA-LFS detected as low as 1 CFU of *Listeria monocytogenes* per reaction without DNA purification [51]. LAMP-LFS has also been used to detect *Salmonella* in milk, pork, beef, and chicken samples at 144 CFU/g or mL, without an enrichment step [50]. These findings collectively demonstrate that NAA-LFS assays, including PCR-LFS, offer highly sensitive detection of target organisms. However, a unique advantage of our PCR-LFS is its ability to detect both the bacterial species (*E. coli* or *Salmonella* spp.) and the antimicrobial resistance gene *bla*_CTX-M_ in a single reaction.

Our current study demonstrated high validity of the dPCR-LFS assay in terms of accuracy, sensitivity, specificity, positive predictive value, and negative predictive value when tested with pure bacterial cultures. Although the assay shows promise for direct application to clinical or environmental samples, further validation using reference methods is necessary. One limitation of the current dPCR-LFS is its ability to detect only two targets per reaction, due to constraints in the availability of label molecules such as biotin and digoxigenin. A triplex format that includes *E. coli*-specific, *Salmonella*-specific, and *bla*_CTX-M_ targets with distinct labels would improve efficiency and reduce cost. Therefore, future development is warranted. Moreover, while the assay was validated using pure isolates, its application to direct specimens remains a key challenge for further research.

## 4. Materials and Methods

### 4.1. Bacteria

*E. coli* and *Salmonella* strains harboring *bla*_CTX-M_, as well as other Enterobacterales species, are listed in Table 1. The 287 *E. coli* strains were isolated from humans (*n* = 162), ready-to-eat foods (*n* = 50), meats (*n* = 40), and environmental water (*n* = 35). A total of 95 *Salmonella* strains were isolated from humans (*n* = 50), ready-to-eat foods (*n* = 15), and meats (*n* = 30). Other Enterobacterales species (Table 1) were isolated from humans (*n* = 118) and environmental sources (*n* = 100). In addition, various non-Enterobacterales species were included to evaluate potential nonspecific reactions. These strains included: *Streptococcus pneumoniae* ATCC33400, *S. anginosus* ATCC33397, *S. pyogenes* SF370, *S. agalactiae* ATCC 13813, *Enterococcus faecalis* ATCC29212, *E. faecium* ATCC10541, *Lactiplantibacillus plantarum* ATCC 43199, *Leuconostoc lactis* ATCC19256, *Micrococcus luteus* ATCC10240, *Bacillus subtilis* ATCC6633, *Staphylococcus aureus* ATCC700698, *Haemophilus influenzae* ATCC10211, *Achromobacter xylosoxidans* ATCC27061, *Pseudomonas aeruginosa* ATCC9027, *Acinetobacter baumannii* ATCC 19606, *Burkholderia cepacia* LMG0122, *Vibrio parahaemolyticus* ATCC17802, *Aeromonas hydrophila* ATCC7966, and *Neisseria flavescens* ATCC13119. All bacterial strains were kept at −80 °C before used in this study.

### 4.2. DNA Extraction

DNA was extracted from cultured bacterial colonies using a simple alkaline lysis method [54]. Briefly, one or two colonies (incubated for 18–24 h) were suspended in 20 µL of lysis buffer containing 0.25% (*v*/*v*) sodium dodecyl sulfate and 0.05 M NaOH, then heated at 95 °C for 15 min. After lysis, samples were briefly centrifuged, and 180 µL of sterile deionized water was added. The crude DNA supernatant was stored at −20 °C and used as the template for PCR.

### 4.3. PCR Reaction

The PCR reaction mixture consisted of 1× PCRBIO HS Taq Mix Red (PCR Biosystems, London, UK) and 0.4 µM of each primer (Table 5). The thermal cycling conditions were as follows: initial denaturation at 95 °C for 2 min; 35 cycles of denaturation at 95 °C for 30 s; annealing at 58 °C for 30 s; and extension at 72 °C for 45 s. *E. coli* ATCC BAA-3303 (carrying *bla_CTX-M-15_*) and *S. enterica* strain SW060-1 (carrying *bla*_CTX-M_) were used as positive controls. *Klebsiella pneumoniae* ATCC 13883 was used as the negative control. PCR products were analyzed by 2% agarose gel electrophoresis (Mupid-ExU, Takara, Japan) in 0.5× TBE buffer for 30 min. Gels were stained with ethidium bromide and visualized under UV light (GeneGenius Bioimaging System; SynGene, Cambridge, UK). PCR product sizes were determined using a 100 bp Plus DNA ladder (GeneRuler™, Thermo Fisher Scientific, Waltham, MA, USA).

### 4.4. Lateral-Flow Strip (LFS)

LFS devices and reagents (K-AmpDetect 2T) were purchased from K.Bio Sciences (Bangkok, Thailand). Each strip had three lines (C, T1, and T2), with T1 and T2 lines immobilized with anti-biotin and anti-digoxigenin, respectively. The LFS reaction was done according to the manufacturer’s protocol. Briefly, strips were immersed in the PCR product for 10–20 s, after which 100 µL (or three drops) of DNA running buffer was added to the application pad. Results were observed within 2–10 min. The presence of both test lines (T1, T2, or both) and the control line (C) indicated a positive result. The appearance of only the control line indicated a negative result.

### 4.5. Confirmation of PCR Products by DNA Sequencing

To confirm the dPCR-LFS results, PCR products from strains testing positive for *E. coli*, *Salmonella*, or *bla*_CTX-M_ were subjected to Sanger DNA sequencing using the respective forward and reverse primers without labelling molecules (Table 5). Sequences were analyzed using BLASTN (https://blast.ncbi.nlm.nih.gov/Blast.cgi; accessed on 5 June 2025).

### 4.6. Detection Limit

The detection limit of the duplex PCR was evaluated using *bla*_CTX-M_-harboring *E. coli* and *Salmonella* strains. Serial 10-fold dilutions were prepared from an initial DNA concentration of 72 ng/µL for *E. coli* and 105 ng/µL for *Salmonella*, based on OD_260_ readings. The detection limit was defined as the highest dilution at which a positive result was observed. Each test was performed in triplicate.

### 4.7. Artificially Spiked Raw Pork Mince Sample

Raw pork mince confirmed to be free of *bla*_CTX-M_-harboring *E. coli* and *Salmonella* by conventional culture and PCR methods was used for spiking experiments. Pure colonies of *E. coli* ATCC BAA-3303 and *S. enterica* strain SW060-1 were suspended in 1 mL of sterile saline, vortexed for 30 s, and adjusted to a turbidity of 1.00 using a turbidimeter. Serial 10-fold dilutions were prepared. Then, 10 g of pork mince was mixed with 99 mL of buffered peptone water (BPW; Oxoid, Basingstoke, UK) and inoculated with 1 mL of the appropriate dilution. One uninoculated sample served as a negative control. After inoculation, samples were stomached for 1 min. One milliliter of the homogenate was subjected to DNA extraction using ZymoBIOMICS DNA Kits (Zymo Research, Tustin, CA, USA) following the manufacturer’s instructions. Each dilution was tested in duplicate. Colony-forming units (CFU) were determined by plating on MacConkey agar for *E. coli* and XLD agar for *Salmonella* (Oxoid, Basingstoke, UK).

### 4.8. Statistical Analysis

Diagnostic performance measures—including sensitivity, specificity, and accuracy—were calculated [58]. The kappa statistic was used to evaluate interrater agreement [59]. Fisher’s exact test (*p* < 0.01 considered significant) was performed using GraphPad tools (https://www.graphpad.com/quickcalcs/, accessed on 5 June 2025) [60,61]. McFadden’s R^2^ was used to estimate model fit [62]. Receiver operating characteristic (ROC) curve analysis was performed using a 2 × 2 contingency table comparing PCR results with genotypic/phenotypic data [63]. Sensitivity and 1 − specificity were plotted to generate the ROC curve. All measurements were done in triplicate. A fixed SD of 0.02 was applied to illustrate variability, similar to approaches used in simulations or illustrative ROC analyses. A dotted reference line indicating ideal performance (100% sensitivity and specificity) was added as a benchmark. All analyses and visualizations were performed using Stata version 17.0.

### 4.9. Data Availability

Nucleotide sequence of *bla*_CTX-M_ is available in the GenBank databases under the accession numbers PV809620-PV809640.

## 5. Conclusions

The dPCR-LFS assay in this study demonstrated high diagnostic performance for detecting *E. coli* and *Salmonella* strains harboring *bla*_CTX-M_ from pure cultures. However, its application to direct specimens requires further validation. This method is rapid and user-friendly, offering simple interpretation without the need for gel electrophoresis. It holds significant potential for application in AMR surveillance across both human and non-human sectors.

## Figures and Tables

**Table 1:** Evaluation of dPCR-LFS for detection of *bla*_CTX-M_ in *E. coli*, *Salmonella*, and other Enterobacterales.

Species	N	Antimicrobial-Resistant Genotypes/Phenotypes	Ciprofloxacin Disk Diffusion	ESBL-Producing	PCR-LFS	
					Species	*bla*_CTX-M_
*E. coli* (N = 287)	100	*bla*_CTX-M_/*bla*_TEM_	R	+	*+*	+
	100	*bla*_CTX-M_/*bla*_NDM_/*bla*_OXA-48-like_ (CRE)	R	+	*+*	+
	7	*bla*_NDM_ (CRE)	R	−	+	−
	5	*bla*_OXA-48-like_ (CRE)	R	−	+	−
	25	*bla*_TEM_/*bla*_SHV_	ND	−	+	−
	10	*bla*_TEM_/*bla*_SHV_	ND	+	+	−
	30	Non-cephalosporins resistance	ND	−	+	−
	10	*bla*_TEM_	ND	−	+	−
*Salmonella* sp. (N = 95)	30	*bla*_CTX-M_, Ciprofloxacin-susceptible	S	+	+	+
	50	Ciprofloxacin-resistance	R	−	+	−
	15	Cephalosporins and Ciprofloxacin-susceptible	S	−	+	−
*Klebsiella pneumoniae* (N = 95)	45	*bla*_CTX-M_/*bla*_SHV_	ND	+	−	+
	25	*bla*_CTX-M_/*bla*_NDM_/*bla*_OXA-48-like_ (CRE)	R	+	−	+
	5	*bla*_NDM_/*bla*_OXA-48-like_ (CRE)	R	−	−	−
	12	*bla*_TEM_/*bla*_SHV_	ND	+	−	−
	8	*bla*_SHV_	ND	+	−	−
*Klebsiella oxytoca*	10	*bla*_CTX-M_/*bla*_SHV_	ND	+	−	+
*Klebsiella aerogenes*	5	*bla*_CTX-M_/*bla*_SHV_	ND	+	−	+
*Enterobacter asburiae*	10	*bla*_CTX-M_/*bla*_NDM_/*bla*_OXA-48-like_ (CRE)	R	+	−	+
*Enterobacter cloacae* complex (N = 25)	10	*bla*_CTX-M_	ND	+	−	+
	10	*bla*_CTX-M_/*bla*_NDM_/*bla*_OXA-48-like_ (CRE)	R	+	−	+
	5	*bla*_NDM_/*bla*_OXA-48-like_ (CRE)	R	−	−	−
*Citrobacter freundii* (N = 28)	6	*bla*_CTX-M_	ND	+	−	+
	14	*bla*_CTX-M_/*bla*_NDM_/*bla*_OXA-48-like_ (CRE)	ND	+	−	+
	8	Cephalosporins susceptible	ND	−	−	−
*Proteus mirabilis*	12	*bla*_CTX-M_	ND	+	−	+
*Proteus vulgaris*	3	none	ND	−	−	−
*Cronobacter sakazakii*	5	none	ND	−	−	−
*Shigella sonnei*	2	none	ND	−	−	−
*Edwardsiella tarda*	2	none	ND	−	−	−
*Morganella morganii*	6	none	ND	−	−	−
*Serratia marcescens*	15	none	ND	−	−	−
Total	600					

**Table 2:** Diagnostic performance of dPCR-LFS in detecting *E. coli* and *Salmonella* harboring *bla*_CTX-M_.

PCR-LFS	Genotypic/Phenotypic Results		*p*-Value
	Positive	Negative	
Positive	200 (*E. coli*)/30 (*Salmonella*)	0	<0.001
Negative	0	71	
Total	230	71	

Accuracy = 100% (95% CI = 98.78% to 100.00%); Sensitivity = 100% (95% CI = 98.41% to 100.00%); Specificity = 100% (95% CI = 94.94% to 100.00%); PPV = 100% (95% CI = 98.41% to 100.00%); NPV = 100% (95% CI = 94.94% to 100.00%); *κ* = 1; R^2^ = 0.92952.

**Table 3:** Diagnostic performance of dPCR-LFS in detecting *E. coli* and *Salmonella* lacking *bla*_CTX-M_.

**PCR-LFS**	**Genotypic/Phenotypic Results**		***p*-Value**
	**Positive**	**Negative**	
Positive	287 (*E. coli*)/95 (*Salmonella*)	0	<0.001
Negative	0	218	
Total	382	218	

Accuracy = 100% (95% CI = 99.39% to 100.00%); Sensitivity = 100% (95% CI = 99.04% to 100.00%); Specificity = 100% (95% CI = 98.32% to 100.00%); PPV = 100% (95% CI = 99.04% to 100.00%); NPV = 100% (95% CI = 98.32% to 100.00%); *κ* = 1; R^2^ = 0.96621.

**Table 4:** Diagnostic performance of dPCR-LFS for detection of *bla*_CTX-M_ among Enterobacterales.

PCR-LFS	Genotypic/Phenotypic Results		*p*-Value
	Positive	Negative	
Positive	377	0	<0.001
Negative	0	223	
Total	377	223	

Accuracy = 100% (95% CI = 99.39% to 100.00%); Sensitivity = 100% (95% CI = 99.03% to 100.00%); Specificity = 100% (95% CI = 98.36% to 100.00%); PPV = 100% (95% CI = 99.03% to 100.00%); NPV = 100% (95% CI = 98.36% to 100.00%); *κ* = 1; R^2^ = 0.96642.

**Table 5:** Primer used in this study.

Primer Name	Sequence (5′–3′)	Product Size (bp)	Target	PCR Set	Reference
uidA-F	5′-FITC-AAAACGGCAAGCAAAAGCAG	147	*E. coli*	1	[55]
uidA-R	5′-Digoxigenin-ACGCGTGGTTAACAGTCTTGCG				
CTX-M-U1	5′-FITC-ATGTGCAGYACCAGTAARGTKATGGC	594	*bla*_CTX-M_		[35]
CTX-M-U2	5′-Biotin-TGGGTRAARTARGTSACCAGAAYCAGCGG				
invA-F	5′-FITC-GAGGAAAAAGAAGGGTCG	780	*Salmonella* spp.	2	[56]
invA-R	5′-Biotin-CTCAACTTCAGCAGATACCA				
CTX-M-F	5′-FITC-TTTGCGATGTGCAGTACCAGTAA	544	*bla*_CTX-M_		[57]
CTX-M-R	5′-Digoxigenin-CGATATCGTTGGTGGTGCCATA				

## Data Availability

Data are contained within the article.
